# Dose–response relationship between device-measured physical activity and incident type 2 diabetes: findings from the UK Biobank prospective cohort study

**DOI:** 10.1186/s12916-023-02851-5

**Published:** 2023-05-24

**Authors:** Jirapitcha Boonpor, Solange Parra-Soto, Fanny Petermann-Rocha, Nathan Lynskey, Verónica Cabanas-Sánchez, Naveed Sattar, Jason M. R. Gill, Paul Welsh, Jill P. Pell, Stuart R. Gray, Frederick K. Ho, Carlos Celis-Morales

**Affiliations:** 1 School of Cardiovascular and Metabolic Health, University of Glasgow, Glasgow, UK; 2 Faculty of Public Health, Kasetsart University, Chalermphrakiat Sakon Nakhon Province Campus, Sakon Nakhon, Thailand; 3 Department of Nutrition and Public Health, Universidad del Bío-Bío, Chillan, Chile; 4 Facultad de Medicina, Universidad Diego Portales, Santiago, Chile; 5 IMDEA Food Institute, CEI UAM+CSIC, Madrid, Spain; 6 School of Health and Wellbeing, University of Glasgow, Glasgow, UK; 7 Human Performance Lab, Education, Physical Activity and Health Research Unit, Universidad Católica del Maule, Talca, Chile

**Keywords:** Accelerometer, Obesity, Physical activity, Type 2 diabetes mellitus

## Abstract

**Background:**

Most studies investigating the association between physical activity (PA) and the risk of type 2 diabetes are derived from self-reported questionnaires, with limited evidence using device-based measurements. Therefore, this study aimed to investigate the dose–response relationship between device-measured PA and incident type 2 diabetes.

**Methods:**

This prospective cohort study included 40,431 participants of the UK Biobank. Wrist-worn accelerometers were used to estimate total, light, moderate, vigorous and moderate-to-vigorous PA. The associations between PA and incident type 2 diabetes were analysed using Cox-proportional hazard models. The mediating role of body mass index (BMI) was tested under a causal counterfactual framework.

**Results:**

The median follow-up period was 6.3 years (IQR: 5.7–6.8), with 591 participants developing type 2 diabetes. Compared to those achieving < 150 min/week of moderate PA, people achieving 150–300, 300–600 and > 600 min/week were at 49% (95% CI 62–32%), 62% (95% CI 71–50%) and 71% (95% CI 80–59%) lower risk of type 2 diabetes, respectively. For vigorous PA, compared to those achieving < 25 min/week, individuals achieving 25–50, 50–75 and > 75 min/week were at 38% (95% CI 48–33%), 48% (95% CI 64–23%) and 64% (95% CI 78–42%) lower type 2 diabetes risk, respectively. Twelve per cent and 20% of the associations between vigorous and moderate PA and type 2 diabetes were mediated by lower BMI, respectively.

**Conclusions:**

PA has clear dose-response relationship with a lower risk of type 2 diabetes. Our findings support the current aerobic PA recommendations but suggest that additional PA beyond the recommendations is associated with even greater risk reduction.

**Trial registration:**

The UK Biobank study was approved by the North West Multi-Centre Research Ethics Committee (Ref 11/NW/0382 on June 17, 2011).

**Supplementary Information:**

The online version contains supplementary material available at 10.1186/s12916-023-02851-5.

## Background

Type 2 diabetes is a common condition, with a growing prevalence worldwide [1, 2], which is associated with an increased risk of numerous adverse health outcomes, such as neuropathy, nephropathy, retinopathy and cardiovascular disease [1, 2]. The identification of modifiable factors associated with the development of type 2 diabetes is of the utmost importance to help identify those at elevated risk and develop strategies to reduce the likelihood of developing this disease. Although obesity is a major risk factor for type 2 diabetes [3], other physical activity-related factors, such as cardiorespiratory fitness and muscle strength, are also associated with the development of type 2 diabetes [4–6].

Physical activity (PA) has been shown to both acutely and chronically improve insulin sensitivity [7] and is included as part of diabetes prevention and treatment interventions, alongside dietary advice [8]. The combination of these factors has resulted in the successful implementation of diabetes intervention programmes, such as the Diabetes Prevention Programme [8]. However, given the well-established importance of weight loss for diabetes prevention, few studies investigated to what extent the association between PA and type 2 diabetes is due to weight control.

We are currently reliant on cohort studies to establish evidence of a potentially causal association between PA with type 2 diabetes, such as demonstrating evidence of a dose-response relationship. To date, most of these studies have used self-reported questionnaires which are prone to recall bias [9] and obscure the true magnitude of associations between PA and type 2 diabetes [10]. Previous studies on all-cause mortality have demonstrated that PA risk reduction estimates based on device measurements are double those derived from questionnaires [11]. Few studies have used device-measured PA on type 2 diabetes outcomes. Those studies were conducted on small study populations (*n* < 8000) of predominantly older adults [12–14] using pedometers to estimate overall step counts. While such data is important, pedometers cannot investigate the associations for different intensities of PA, something we can achieve with accelerometers. Only two studies [13, 15] have been conducted on middle-aged adults using PA assessed by accelerometry.

However, these studies reported the association of only moderate-to-vigorous PA with type 2 diabetes risk, which is limited as increasing evidence highlights the potential importance of light PA. Neither of these studies investigated whether current PA recommendations [16] were supported by evidence derived from device-measured PA for type 2 diabetes prevention. Therefore, the current study aimed to investigate the dose–response relationship between intensity-specific physical activity, quantified by accelerometry, and incident type 2 diabetes.

## Methods

### Population

The UK Biobank recruited over 500,000 participants between 2006 and 2010 (5.5% response rate) from the general population [17]. At the baseline assessment (between 2006 and 2010), participants attended one of 22 assessment centres across Scotland, England and Wales [18, 19]. Participants completed electronic consent and touch screen questionnaires and had physical measurements taken, and biological samples collected, as described elsewhere [18, 19]. Device-based physical activity was assessed in 96,519 participants between 2013 and 2015. Therefore, the end date of the accelerometer wear time was used as the start date for the Cox regression analysis which means that type 2 diabetes cases diagnosed before this date were excluded. Participants with prevalent type 1, type 2 diabetes (*n* = 1534) or undiagnosed diabetes (HbA1c ≥ 48 mmol/mol) (*n* = 206) at the baseline assessment as well as 132 missing relevant covariates were also excluded (Additional file 1: Fig. S1). After applying these exclusions, 40,431 participants had data available on device-measured PA, incident type 2 diabetes and relevant covariates. More information about the UK Biobank protocol can be found online (http://www.ukbiobank.ac.uk).

### Physical activity

Axivity AX3 wrist-worn triaxial accelerometers were used to collect objectively measured PA [20]. The device was worn on the dominant wrist over a period of 7 days at 100 Hz. Of the 103,681 participants who agreed to wear accelerometers, 7162 were excluded due to insufficient wear time (< 72 h wear), missing data or poor device calibration, resulting in 96,519 participants being eligible for inclusion in the analyses. However, only 40,431 participants with data available for device-measured PA, diabetes incidence and covariates were included in the current study (Additional file 1: Fig. S1). More details about the data collection and processing can be found elsewhere [20].

The average vector magnitude in milligravities (mg) was used to estimate the total volume of PA. Minutes per week (min/week) of light and moderate and vigorous PA were determined from the time spent at 30–125 mg, 125–400 mg and > 400 mg of acceleration, respectively [21, 22]. In accordance with the current PA recommendations [16], the following categories for moderate (< 150, 150–299, 300–599 and ≥ 600 min/week) and vigorous (< 25, 25–49, 50–74 and ≥ 75 min/week) PA were derived. Moderate-to-vigorous PA (MVPA) was estimated from the sum of moderate PA and vigorous PA × 2 and expressed in minutes per week. Total PA was derived from the sum of light (assumed on average 3 METs), moderate (average 4 METs) and vigorous PA (average 8 METs) and expressed as MET-minutes per week. As the intensity-weighted domains of PA could produce imprecise risk estimates, MVPA and total PA were also presented as unweighted minutes per week and as total acceleration counts for total PA.

### Incident type 2 diabetes

Prevalent type 1 and type 2 diabetes were ascertained from self-report and from HbA1c concentrations ≥ 6.5% at baseline (undiagnosed diabetes). Incident type 2 diabetes was ascertained from linkage to primary care records and hospital inpatient records. Records were available up to March 2021, and detailed procedures can be found in the UK Biobank online resource (http://www.ukbiobank.ac.uk/). Participants with at least one new record of type 2 diabetes, from either primary care or hospital inpatient data, were defined as having incident type 2 diabetes. Records of type 2 diabetes at primary and secondary diagnoses were defined as International classification of diseases, 10th revision (ICD-10) code E11 or equivalent READ codes, mapped using UK Biobank’s look-up table (https://biobank.ndph.ox.ac.uk/showcase/field.cgi?id=41270).

### Covariates

The covariates included were assessed at the baseline assessment visit (between 2006 and 2010). Age was calculated from dates of birth and baseline assessment date; ethnicity was self-reported and categorized as White, South Asian, Black or other/mixed backgrounds. The deprivation index, an area-based measure of socioeconomic status, was derived from the postal code of residence using the Townsend deprivation score [23]. Education achievement was self-reported at baseline. Alcohol intake was self-reported and categorized as daily or almost daily, 3–4 times a week, once or twice a week, 1–3 times a month, special occasions only and never. Smoking status was self-reported as never, former or current smoker. BMI was measured at baseline, and it was calculated as weight (in kilograms (kg)) divided by the square of height (in meters (m)), and the WHO criteria were applied to categorise participants into underweight (BMI < 18.5 kg/m^2^), normal (18.5 to 24.9 kg/m^2^), overweight (25.0 to 29.9 kg/m^2^) or obese (≥ 30.0 kg/m^2^) [24]. Waist circumference (WC) was measured midway between the lowest rib margin and the iliac crest, in a horizontal plane, using a non-elastic SECA 200 tape measure. WC ≥ 88 cm for women and ≥ 102 cm for men was used to define central obesity. Additional details about these measurements can be found in the UK Biobank online protocol [25].

### Ethical approval

The UK Biobank study was approved by the North West Multi-Centre Research Ethics Committee (Ref 11/NW/0382 on June 17, 2011), and all participants provided written informed consent to participate in the UK Biobank study. The study protocol is available online. This research has been conducted using the UK Biobank resource under application number 7155.

### Statistical analyses

Continuous variables were expressed as means and standard deviations (SD) and categorical variables as frequencies and percentages by categories of total PA. Cox-proportional hazard models were used to investigate the associations between PA domains and incident type 2 diabetes, with years of follow-up as the timeline variable. The end date of the accelerometer wearing time was used as the start of the follow-up. The results were reported as hazard ratios (HRs) together with 95% confidence intervals (CIs). Incident cases, total person-year per 10,000 participants and incident rate per 100,000 person-year were also estimated. The analyses included participants who had accelerometer, primary care data and hospital inpatient records available but excluded those with prevalent type 1, type 2 (*n* = 1534) or undiagnosed diabetes (HbA1c ≥ 6.5%) (*n* = 206) at baseline assessment.

We used categorized variables and nonlinear analyses to investigate the associations of total PA and intensity-specific PA domains with incident type 2 diabetes. Firstly, PA exposures were fitted into the model as categories of moderate and vigorous PA and MVPA. Next, nonlinear associations between PA domains and incident type 2 diabetes were investigated using penalised cubic splines fitted in Cox proportional hazard models. The penalised spline is a variation of the basis spline, which is not as sensitive to knot numbers and placements as restricted cubic splines [26]. Nonlinearity in exposure-outcome relationships was tested by likelihood ratio tests comparing models with PA splines and models with linear PA terms. The proportional hazard assumption was checked using Schoenfeld residuals. A 2-year landmark analysis was applied to minimise reverse causation.

The association between PA and incident type 2 diabetes was adjusted for covariates measured at the baseline assessment (2006–2010). These covariates were age, sex, deprivation index, ethnicity, education, smoking status and alcohol intake. These covariates are likely confounders (i.e. common causes for PA and type 2 diabetes) and thus were our main model. We conducted a sensitivity analysis by mutually adjusting the PA intensities (light, moderate and vigorous). In addition, BMI categories were also included as a covariate in a sensitivity analysis model. This model was only used for examining the non-linear association between PA and type 2 diabetes as BMI is a likely mediator. The joint association between moderate and vigorous PA was created by using the risk matrix. Another sensitivity spline analysis was conducted using time spent on total PA and MVPA without weighting for intensity as those might not be precise.

As BMI is likely to mediate PA and type 2 diabetes risk, we performed a causal counterfactual framework analysis [27]. Adjusting for all the confounders included in the main model, type 2 diabetes was regressed by PA and BMI (outcome model), and BMI was regressed by PA (mediator model). The outcome and mediator models were then combined to compute the natural indirect effect (NIE) and total effect (TE) for each participant, which was then averaged. Quasi-Bayesian estimation with 1000 iterations was used for estimating the 95% CI and *p*-values of the NIE and TE. The mediation proportion was calculated as NIE/TE. Sensitivity analysis for mediation was conducted by using WC (normal vs central obesity) instead of BMI.

Rate advancement periods [28] were used to estimate the number of additional chronologic years that would be required to yield the equivalent risk rate of incident type 2 diabetes among individuals who reported the lowest PA compared to those who reported higher levels of PA, as described elsewhere [29].

Preventable fractions for the study population (PFP) [30] were calculated to estimate the proportions of all incident type 2 diabetes cases that could have been prevented if the individuals in different PA categories were as active as the most active group, assuming that the associations were causal.

Statistical analyses were performed using the statistical software STATA 17 (StataCorp LP) and R v4.0.2. *p*-values < 0.05 were regarded as statistically significant.

## Results

Of the 502,458 participants who were enrolled in UK Biobank, 40,431 participants with data available for accelerometry-measured PA, incident type 2 diabetes and covariates were included in this study (Additional file 1: Fig. S1). The median follow-up period was 6.3 years (interquartile range: IQR: 5.7–6.8). Over the follow-up period, 591 participants were diagnosed with type 2 diabetes (245 women and 346 men).

Table 1 presents the general characteristics of the participants, categorized by total PA quartiles. Compared to those in the highest quartile of total PA, individuals with the lowest total PA were older and had similar educational qualifications but higher area deprivation. They had higher BMI and WC and were more likely to be current smokers but never drink alcohol.

**Table 1:** Cohort characteristics by total physical activity quartile

Characteristics	Lowest	Low-middle	Middle-high	Highest
Women	4534 (46.3)	5723 (56.0)	6269 (61.4)	6755 (66.2)
Men	5263 (53.7)	4502 (44.0)	3935 (38.6)	3450 (33.8)
Age, years (mean, SD)	57.4 ± 7.7	56.3 ± 7.8	55.7 ± 7.7	54.8 ± 7.7
**Ethnicity, *n* (%)**				
White	9548 (98.7)	9967 (98.7)	9967 (98.8)	9900 (98.5)
South Asian	82 (0.9)	81 (0.8)	72 (0.7)	90 (0.9)
Black	41 (0.4)	54 (0.5)	46 (0.5)	60 (0.6)
**Education qualifications, *n* (%)**				
College or university degree	4705 (53.9)	5134 (55.0)	5014 (53.4)	4707 (50.5)
A levels/AS levels or equivalent	1222 (14.0)	1261 (13.5)	1377 (14.7)	1275 (13.7)
O levels/GCSEs or equivalent	1874 (21.5)	2036 (21.8)	2108 (22.5)	2298 (24.6)
CSEs or equivalent/NVQ or HND or HNC	935 (10.7)	905 (9.7)	887 (9.5)	1050 (11.3)
**Townsend Deprivation Index, *n* (%)**				
Lower deprivation	3534 (36.1)	3870 (37.9)	4003 (39.2)	3860 (37.8)
Middle deprivation	3394 (34.6)	3660 (35.8)	3619 (35.5)	3577 (35.1)
Higher deprivation	2869 (29.3)	2695 (26.4)	2582 (25.3)	2768 (27.1)
**Alcohol intake, *n* (%)**				
Daily or almost daily	2112 (21.6)	2304 (22.5)	2314 (22.7)	2279 (22.3)
3–4 times a week	2394 (24.4)	2773 (27.1)	2815 (27.6)	2743 (26.9)
Once or twice a week	2503 (25.6)	2602 (25.5)	2636 (25.8)	2544 (24.9)
1–3 times a month	1188 (12.1)	1128 (11.0)	1047 (10.3)	1073 (10.5)
Special occasions only	992 (10.1)	923 (9.0)	851 (8.3)	969 (9.5)
Never	608 (6.2)	495 (4.8)	541 (5.3)	597 (5.9)
**Smoking status, *n* (%)**				
Never	5368 (54.9)	5958 (58.4)	6005 (58.9)	6061 (59.5)
Previous	3551 (36.3)	3609 (35.4)	3577 (35.1)	3498 (34.4)
Current	855 (8.8)	638 (6.3)	612 (6.0)	620 (6.1)
**Physical activity**				
Total physical activity, min/day	263.3 ± 38.8	338.7 ± 15.6	392.2 ± 16.3	476.4 ± 47.4
Light physical activity, min/day	220.1 ± 33.9	275.2 ± 24.3	312.1 ± 27.8	366.1 ± 42.8
Moderate physical activity, min/day	40.8 ± 18.2	59.6 ± 19.9	75.1 ± 23.2	103.1 ± 34.6
Vigorous physical activity, min/day	2.3 ± 3.7	3.9 ± 5.2	5.1 ± 6.2	7.2 ± 7.8
MVPA, METs/min/week	1273.9 ± 616.8	1886.2 ± 705.4	2388.9 ± 831.6	3292.0 ± 1206.5
**Adiposity**				
BMI, kg/m^2^ (mean, SD)	27.9 ± 4.8	26.7 ± 4.2	26.1 ± 4.1	25.4 ± 3.9
Waist circumference, cm (mean, SD)	92.3 ± 12.8	88.2 ± 12.1	86.1 ± 11.9	83.7 ± 11.7
**BMI category, *n* (%)**				
Underweight (< 18.5 kg/m^2^)	32 (0.3)	40 (0.4)	53 (0.5)	80 (0.8)
Normal (18.5–24.9 kg/m^2^)	2751 (28.1)	3818 (37.3)	4437 (43.5)	5108 (50.1)
Overweight (25–29.9 kg/m^2^)	4321 (44.1)	4469 (43.7)	4187 (41.0)	3828 (37.5)
Obese (≥ 30.0 kg/m^2^)	2693 (27.5)	1898 (18.6)	1527 (15.0)	1189 (11.7)

Data are presented as mean and standard deviation (SD) for continuous variables and as frequency and % for categorical variables

*A levels/AS levels*, advanced/advanced subsidiary levels; *BMI* Body mass index, *CSE* certificate of secondary education, *GCSE* General certificate of secondary education, *HNC* Higher national certificate, *HND* Higher national diploma, *MET* Metabolic equivalent, *NVQ* National vocational qualification, *O levels*, Ordinary levels, *PA* Physical activity

**Fig. 1:**
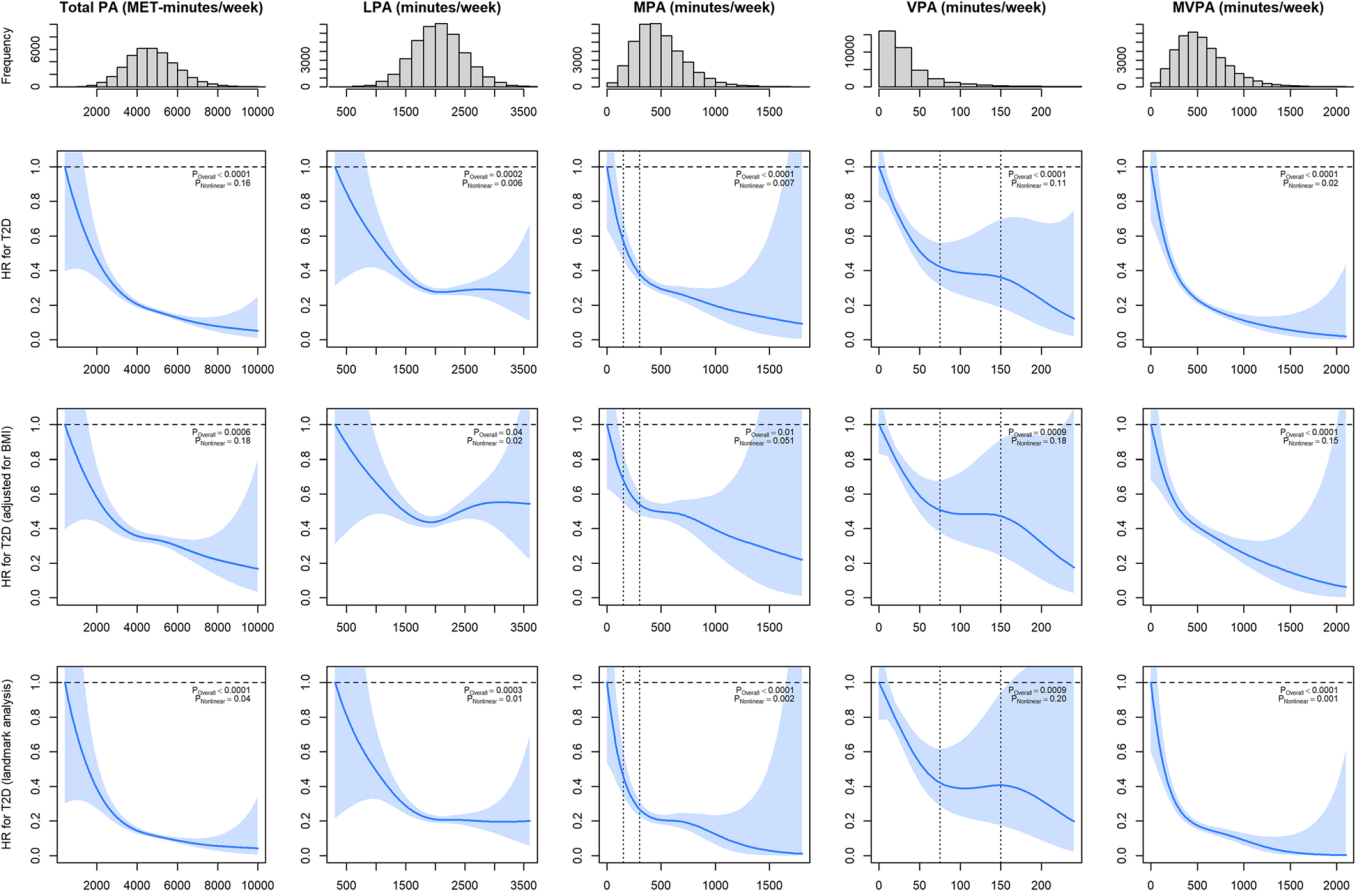
Non-penalised cubic splines for the association between physical activity domains and incident type 2 diabetes. Data are presented as hazard ratios (HR) and their 95% CI. The top row of the panel was adjusted for age, sex, deprivation, education, ethnicity, alcohol intake and smoking status. The second row was additionally adjusted for BMI. The bottom row was adjusted for the same covariates as the middle row but include a 2-year landmark analysis. Vertical dotted lines represent the current PA recommendations for moderate and vigorous PA. LPA, light physical activity; MPA, moderate physical activity; MVPA, moderate-vigorous physical activity; PA, physical activity; T2D, type 2 diabetes; VPA, vigorous physical activity. MVPA is the sum of time spent on MPA and VPA × 2

The associations between PA domains and incident type 2 diabetes adjusted for sociodemographic and lifestyle factors (main model) are presented in Table 2 and Fig. 1. Compared to participants who performed < 150 min/week of moderate PA, those who performed between 150–299, 300–599 and ≥ 600 min/week had a 49%, 62% and 71% lower risk of incident type 2 diabetes, respectively (Table 2). The splines for moderate PA showed that type 2 diabetes risk decreased sharply with increasing moderate PA up to the level of 300 min/week, with risk reduction levelling off thereafter (Fig. 1). For vigorous PA, the results suggest a 38%, 48% and 64% lower risk of incident type 2 diabetes for people achieving 25–49, 50–74 and ≥ 75 min/week, respectively, compared to those achieving < 25 min/week (Table 2). The splines for vigorous PA showed that the risk of incident type 2 diabetes appears to lower sharply with greater vigorous PA up to 75 min/week, plateaued between 75 and 150 min/week, then reduced further above 150 min/week (Fig. 1). For light PA, the lowest risk was observed around 1500 min/week, then plateaued thereafter (Fig. 1). The dose–response relationships for both total PA expressed in MET-min/week and MVPA expressed in minutes/week were similar, with the risk of incident type 2 diabetes decreasing sharply up to the level of 3000 MET-min/week for total PA and 400 min/week of MVPA with risk reduction levelling off thereafter.

**Table 2:** Risk and the preventable fraction of incident type 2 diabetes by categories of moderate and vigorous PA

	**Prevalence in the study sample (%)**	**Incident cases**	**Total person-years (10,000)**	**Incident rate per 100,000 person-years**	**HR (95% CI)**	**Rate advancement period**^b^ **(95% CI)**	**Preventable fractions for the population**^a^	
							**% (95% CI)**	**Cumulative % (95% CI)**
**MPA, min/week**								
0 to < 150	3.45	67	0.8	86.8	1.00 (reference)	Reference	6.18 (4.95; 7.23)	6.18 (4.99; 7.25)
150 to < 300	17.29	155	4.0	38.4	0.51 (0.38; 0.68)	16.2 (7.2; 32.7)	9.67 (6.25; 13.42)	15.85 (12.08; 19.43)
300 to < 600	51.88	274	12.3	22.3	0.38 (0.29; 0.50)	23.9 (13.0; 41.9)	11.97 (3.89; 21.75)	27.82 (20.89; 36.35)
≥ 600	27.38	78	6.6	11.9	0.29 (0.20; 0.41)	29.8 (16.7; 54.5)	Reference	Reference
**VPA, min/week**								
0 to < 25	60.05	455	14.1	32.3	1.00 (reference)	Reference	47.42 (41.17; 55.51)	47.42 (40.68; 55.25)
25 to < 50	18.80	68	4.5	15.2	0.62 (0.52; 0.77)	11.5 (4.9; 22.1)	6.01 (2.12; 10.23)	53.43 (47.92; 61.61)
50 to < 75	11.09	32	2.7	12.1	0.52 (0.36; 0.77)	15.7 (4.9; 34.6)	2.26 (− 0.44; 5.07)	55.69 (50.34; 62.72)
≥ 75	10.06	19	2.4	7.8	0.36 (0.22; 0.58)	24.6 (10.2; 51.2)	Reference	Reference
**MVPA, min/week**								
0 to < 150	3.12	61	0.7	84.2	1.00 (reference)	Reference	6.97 (6.09–7.73)	6.97 (6.09–7.73)
150 to < 300	14.11	148	3.6	41.1	0.57 (0.42–0.78)	13.5 (4.6; 29.4)	14.56 (12.50–16.45)	21.53 (19.31–23.70)
300 to < 600	45.59	268	11.5	23.3	0.36 (0.27–0.49)	24.5 (13.3; 44.3)	20.23 (15.65–25.32)	41.76 (38.30–45.76)
≥ 600	37.18	97	7.8	12.4	0.21 (0.150.29)	37.6 (23.2; 64.2)	Reference	Reference

MPA and VPA were mutually adjusted. All analyses were adjusted for age, sex, deprivation, education, ethnicity, alcohol intake and smoking status. BMI (model 2) was not adjusted as this is a likely mediator as confirmed in the mediation analysis

MVPA is the sum of time spent on MPA and VPA × 2

*HR* Hazard ratio, *MPA* Moderate-intensity PA, *MVPA* Moderate to vigorous-intensity PA, *PA* Physical activity, *VPA* Vigorous-intensity PA

^a^Preventable fractions estimated the fractions of all incident type 2 diabetes in the study population that could have been prevented if the individuals in those PA categories were as active as the reference group

^b^Rate advancement period was conducted to estimate the number of additional chronologic years that would be required to yield the equivalent risk rate of type 2 diabetes incidence among individuals who reported the higher PA compared to those who reported the lowest PA

Sensitivity analyses applying a 2-year landmark did not alter these associations (Fig. 1). However, when the analysis for MVPA was adjusted for BMI, the shape of the association with incident type 2 diabetes becomes linear (Fig. 1). When light, moderate and vigorous PA were mutually adjusted, the shape of associations remained similar (Additional file 1: Fig. S2). However, when BMI was added as a covariate, the magnitude of associations was slightly attenuated but remained significant (Fig. 1). Using unweighted PA variables resulted in similar findings (Additional file 1: Fig. S3).

Mediation analyses showed that BMI explained up to 23.6%, 19.6% and 12.5% of the associations of total PA, moderate PA and vigorous PA with incident type 2 diabetes, respectively. There was no evidence to support BMI mediating the associations between light PA and type 2 diabetes risk (Additional file 1: Table S1). Similar results were found for WC (as a sensitivity analysis), but WC mediated the associations of total PA with incident type 2 diabetes at ~ 9%, as shown in Additional file 1: Table S1.

**Fig. 2:**
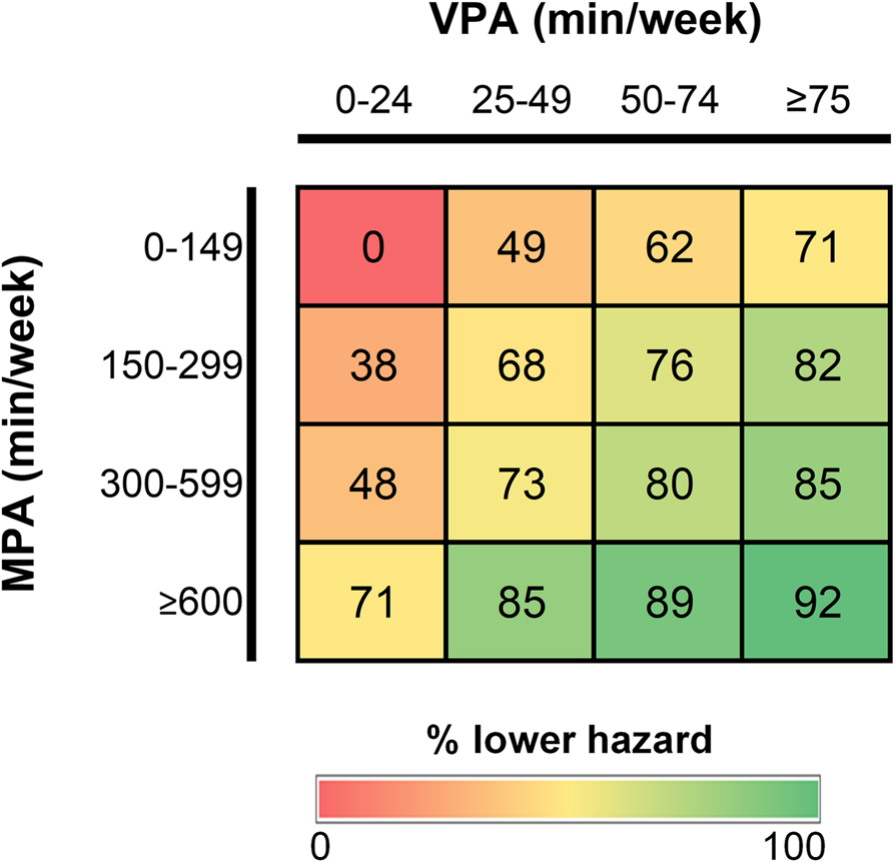
Risk matrix for the joint association of MPA and VPA with incident type 2 diabetes. Estimated in Cox regression adjusted for age, sex, deprivation, education, ethnicity, alcohol intake and smoking status. The numbers presented are the associated reduction in hazard (%) compared with the least active group, based on the hazard ratios (HRs) shown in Table 2. MPA, moderate physical activity; VPA, vigorous physical activity

A risk matrix for the joint associations between moderate and vigorous PA is presented in Fig. 2. Undertaking a lower amount of moderate (< 150 min/week) but more than 75 min/week of vigorous PA was associated with a 71% lower risk of incident type 2 diabetes. Similarly, a 71% lower risk of incident type 2 diabetes was observed for those doing little vigorous PA (< 25 min/week) but high amounts of moderate PA (> 600 min/week) (Fig. 2). The lowest (92%) risk was observed in those participants doing > 600 and > 75 min/week of moderate and vigorous PA, respectively, compared to the least active (< 150 and 25 min/week of moderate and vigorous PA) (Fig. 2).

The proportions of type 2 diabetes cases that increased PA could have prevented are presented in Table 2. Assuming causality, 7% of incident type 2 diabetes cases in the study population could have been prevented if all participants had met current aerobic PA recommendations of 150 to 300 min/week of MVPA, and 41.8% could have been prevented if participants had undertaken 600 min/week of MVPA. The rate advancement period analysis revealed that people who do not meet the current PA guidelines (< 150 min/week of MVPA) would experience the equivalent type 2 diabetes risk rate as those who met the recommendation (150–300 min/week) 13.5 years earlier. Compared to those who undertook twice the current PA recommendation (≥600 min/week of MVPA), people who achieved less than 150 min/week experienced a similar type 2 diabetes risk rate 37.6 years earlier (Table 2).

## Discussion

Objectively measured PA—whether of light, moderate or vigorous intensity—had a protective association with the development of type 2 diabetes, with less than one-fifth of the association explained by BMI. Our findings reinforced the importance of individuals meeting current aerobic PA recommendations (150–300 min/week of MVPA) in that adherence to the recommendations would delay type 2 diabetes by 13.5 years and could prevent 7% of type 2 diabetes cases. Indeed, low doses of MVPA were associated with rapid declines in the risk of type 2 diabetes. However, our results also suggested that going beyond current recommendations could produce much greater benefits, even beyond reductions in BMI. The dose–response relationship was observed across the full range of moderate and vigorous PA, with no observable plateau. For example, 41.8% of type 2 diabetes cases potentially be prevented if people perform ≥ 600 equivalent minutes/week of MVPA.

Although there is a large amount of evidence based on self-reported PA that supports an inverse association between PA and type 2 diabetes risk [31], there is very limited evidence from studies using device-measured PA. To date, only two small-scale studies have been conducted using accelerometer-measured PA [13, 15], while another three studies have been based on step count using pedometers in older adults [12, 14, 32]. The Hispanic Community Health Study [15], which included 7280 adults aged between 18 and 74 years and who were followed up for 6 years (871 type 2 diabetes incident cases), reported that MVPA, measured using an Actical accelerometer, was inversely and nonlinearly associated with lower type 2 diabetes risk. Compared to the lowest quartile for MVPA, a similarly lower risk was observed for those in the 2nd, 3rd and 4th (highest) quartile of MVPA, with hazard ratios ranging from 0.76 to 0.67. The authors reported that type 2 diabetes decreased sharply with increasing MVPA up to a level of 30 min/day, but no further benefits were observed beyond this point. Another study assessing the association between PA, measured by Actigraph accelerometers, and type 2 diabetes risk was the Coronary Artery Risk Development in Young Adults (CARDIA) study. This study included 2291 adults aged between 38 and 50 years who were followed up for 5 to 10 years (147 participants developed type 2 diabetes). The authors reported 45% and 30% lower type 2 diabetes for those in the highest compared to the lowest PA tertile [13], for men and women, respectively. Although these studies partially agree with our findings, the association with other PA domains such as light or total PA was not investigated. In addition, none of these studies investigated whether current aerobic PA recommendations were associated with lower type 2 diabetes risk, as we reported in the current study. Furthermore, the authors simply adjusted for BMI rather than estimate how much of the association between PA and type 2 diabetes risk was explained by baseline BMI, which is something we were able to do in the current study.

Other studies have also investigated the association between step count as a proxy of overall PA, and type 2 diabetes risk; however, most, but not all, of them have been conducted in older adults [12–15, 32]. Although these studies cannot be compared directly to our findings due to the different approaches to measuring the exposure, the findings are consistent, showing that a higher number of steps is associated with a lower risk of incident type 2 diabetes. The OPACH study conducted on 3279 older women (mean age 78.9 years) followed-up for 6.9 years (395 developed diabetes) reported that total steps per day were linearly associated with lower type 2 diabetes risk. However, when intensity was estimated, only moderate-intensity, not light-intensity steps, was associated with a lower risk of type 2 diabetes (HR: 0.86 per 2000 steps increment, 95% CI 0.74–1.00) [14]. Other studies reporting similar findings are the Nateglinide and Valsartan in Impaired Glucose Tolerance Outcomes Research (NAVIGATOR) trial. This study reported that the risk of type 2 diabetes was 6% lower per 2000 steps/day, without accounting for intensity [12]. Similarly, the Healthy Ageing Initiative study (HAI), which included 3055 community-dwelling 70-year-old participants (52% women), who were followed up for 2.6 years (81 developed type 2 diabetes), reported a nonlinear inverse association between steps and risk of type 2 diabetes. A steeper decline in the risk of type 2 diabetes was observed from a lower daily step count until around 6000 steps/day, without accounting for intensity [32].

Our study meaningfully extends this literature [33] by showing that lower baseline BMI only partially mediates the association between PA and type 2 diabetes risk. Therefore, the association between PA and the reduction of type 2 diabetes risk is likely due to the variety of other mechanisms through which PA improves glycaemic control [5, 34–37]. Previous data showing the benefits of PA for glycaemic control provides strong support for the association between PA and type 2 diabetes being causal in nature, but this should be confirmed in appropriately designed trials.

Our findings are of important public health relevance as they provide strong evidence using device-measured PA that current recommendations for MVPA are effective for type 2 diabetes risk prevention but that higher PA levels are associated with even greater benefits. We demonstrated that adherence to the recommendations delays the onset of type 2 diabetes and prevents some cases. This has important economic implications given that the global cost of type 2 diabetes is on target to almost double to $2.5 trillion by 2030 [38]. Moreover, our study also provides evidence that type 2 diabetes risk estimates derived from previous self-reported PA studies have underestimated the true magnitude of the associations between PA and type 2 diabetes risk. A meta-analysis of 28 prospective cohort studies estimated that self-reported PA was associated with 26% lower type 2 diabetes risk (95% CI 20–31%) among those who achieved 150 min/week of moderate PA relative to inactive individuals. Achieving twice this amount of PA was associated with a risk reduction of 36% (95% CI 27–46%) [10]. These risk estimates were weaker than the ones reported in the current study, where individuals meeting moderate PA recommendations had a 49% lower risk of type 2 diabetes, while those achieving twice this amount had a 62% lower risk. The differences may be attributable to recall bias when questionnaires are used, but there is also a possibility that this could be explained by the algorithms used to quantify PA. As questionnaires used in previous studies recorded data on PA only if it was performed in 10-min bouts, which is no longer required on the latest PA guidelines [16] and therefore, it was not applied in the current analysis of our accelerometry data.

The strengths of the present study include the large number of participants, which allowed us to explore the dose–response relationship between physical activity and type 2 diabetes risk. An extensive list of confounders, in comparison with previous studies, was also considered. The current study used accelerometers to measure PA. This is a substantial advantage over much of the existing literature as it overcomes the limitations related to recall bias and misclassification from PA questionnaires.

However, the present study is not exempt from limitations. All the covariates in this study were measured earlier than the measurement of PA; therefore, some of them could change between the baseline assessment and the date when the device-based physical activity was measured. The UK Biobank is not representative of the general population of the UK, including sociodemographic, physical, lifestyle and health-related characteristics. Effect size estimates are still generalisable to the broader population, but estimates of cases avoided may be an underestimate of the true figure in the general population due to UK Biobank participants having a healthier lifestyle [39, 40]. In addition, the findings reported in our study could not be generalised to non-white ethnic groups as more the 95% of the participants were White Europeans. We also should consider the health-promoting effect of physical activity assessment by accelerometer, which could influence the physical activity behaviours of participants. Although BMI was used as a proxy of adiposity in our study, other markers such as body fat may explain a higher proportion of the mediation. Reverse causation is another potential limitation of this study, but we attempted to mitigate this risk by conducting a 2-year landmark analysis excluding participants who were recorded as developing diabetes within the first 2 years of follow-up. Additionally, we run correlations between BMI measured at baseline and three additional times during the follow-up (between 2006 and 2019). The correlation across all these time points was very high (*r* ≥ 0.89) suggesting that BMI has been pretty stable during the follow-up. Although we could not rule out reverse causation, the high correlation of BMI across different time points suggested that the bias coming from when BMI was measured may not have a strong bias effect on the mediation analysis (Additional file 1: Table S2).

## Conclusions

The current study provides evidence that all forms of objectively measured PA are associated with lower type 2 diabetes risk in a dose-response relationship. Our findings broadly support current PA guidelines and suggest they could be even more ambitious. Public health policy aiming to increase PA at population levels is important as an adjunct to dietary-induced weight-loss strategies to prevent type 2 diabetes.

## Supplementary Information

**Additional file 1: Table S1.** Mediation analysis between physical activity and incident type 2 diabetes by body mass index and waist circumference. **Table S2.** Pearson correlation coefficients for body mass index at four times points. **Fig. S1.** Flowchart of participants. **Fig. S2.** Non-linear association between physical activity domains and incident type 2 diabetes using mutually adjusted physical activity domains. **Fig. S3.** Non-linear association between unweighted physical activity domains and incident type 2 diabetes.

## Data Availability

The dataset supporting the conclusions of this article is available upon request from the UK Biobank (https://www.ukbiobank.ac.uk/).

## Abbreviations

BMI: Body mass index
CI: Confidence interval
HbA1c: Heamoglobin A1c
HR: Hazard ratio
ICD: International Classification of Diseases
IQR: Interquartile range
MET: Metabolic equivalent of task
MVPA: Moderate-to-vigorous physical activity
NIE: Natural indirect effect
PA: Physical activity
PFP: Preventable fractions for the study population
*r*: Pearson correlation coefficient
TE: Total effect
WC: Waist circumference
