# Supplementary material for: Dose–response relationship between device-measured physical activity and incident type 2 diabetes: findings from the UK Biobank prospective cohort study
Source: BMC Med. 2023 May 24;21:191. doi: 10.1186/s12916-023-02851-5 (PMC10210409; doi:10.1186/s12916-023-02851-5)
Supplement: Supplementary file 1 — Additional file 1: Table S1. Mediation analysis between physical activity and incident type 2 diabetes by body mass index and waist circumference. Table S2. Pearson correlation coefficients for body mass index at four times points. Fig. S1. Flowchart of participants. Fig. S2. Non-linear association between physical activity domains and incident type 2 diabetes using mutually adjusted physical activity domains. Fig. S3. Non-linear association between unweighted physical activity domains and incident type 2 diabetes. [file 12916_2023_2851_MOESM1_ESM.docx]

**ADDITIONAL FILE 1**

**Table S1**. Mediation analysis between physical activity and incident type 2 diabetes by body mass index and waist circumference.

| **Type of physical activity** | **Mediation via BMI** | | **Mediation via WC** | |
| --- | --- | --- | --- | --- |
|  | % of mediation | P-value | % of mediation | P-value |
| Light PA | 6.0 | 0.260 | 2.4 | 0.330 |
| Moderate PA | 19.6 | <0.001 | 20.0 | <0.001 |
| Vigorous PA | 12.5 | <0.001 | 12.5 | <0.001 |
| Total PA | 23.6 | <0.001 | 9.4 | 0.004 |

The analyses were adjusted for age, sex, deprivation, education, ethnicity, alcohol intake, and smoking status.

BMI: body mass index; WC: waist circumference; PA: physical activity

**Table S2**. Pearson correlation coefficients for body mass index at four times points.

|  | Baseline assessment in 2006  N=499,305 | Second assessment in 2012  N=20296 | Third assessment in 2014  N=49720 | Fourth assessment visit 2019  N= 5270 |
| --- | --- | --- | --- | --- |
| Initial assessment | 1.00 |  |  |  |
| First repeat assessment | 0.93 | 1.00 |  |  |
| Imaging visit | 0.89 | 0.94 | 1.00 |  |
| First repeat imaging | 0.90 | 0.89 | 0.94 | 1.00 |

502,458 participants

recruited by the UK Biobank study between 2006 and 2010

**Exclusion**

405,939 participants without valid device-measured PA data

96519 participants

with available device-measured physical activity data

**Exclusion**

53480 participants without linked primary care data or inpatient hospital record

1534 participants due to type 1 or 2 diabetes diagnosed prior to baseline

206 due to unknown diabetes status at baseline

132 due to missing covariates

736 due to incident T2D occurred in the first 2 years of follow-up

40431 participants

with full data available for incident type 2 diabetes, device-measured physical activity and covariates

**Figure S1**. Flowchart of participants.


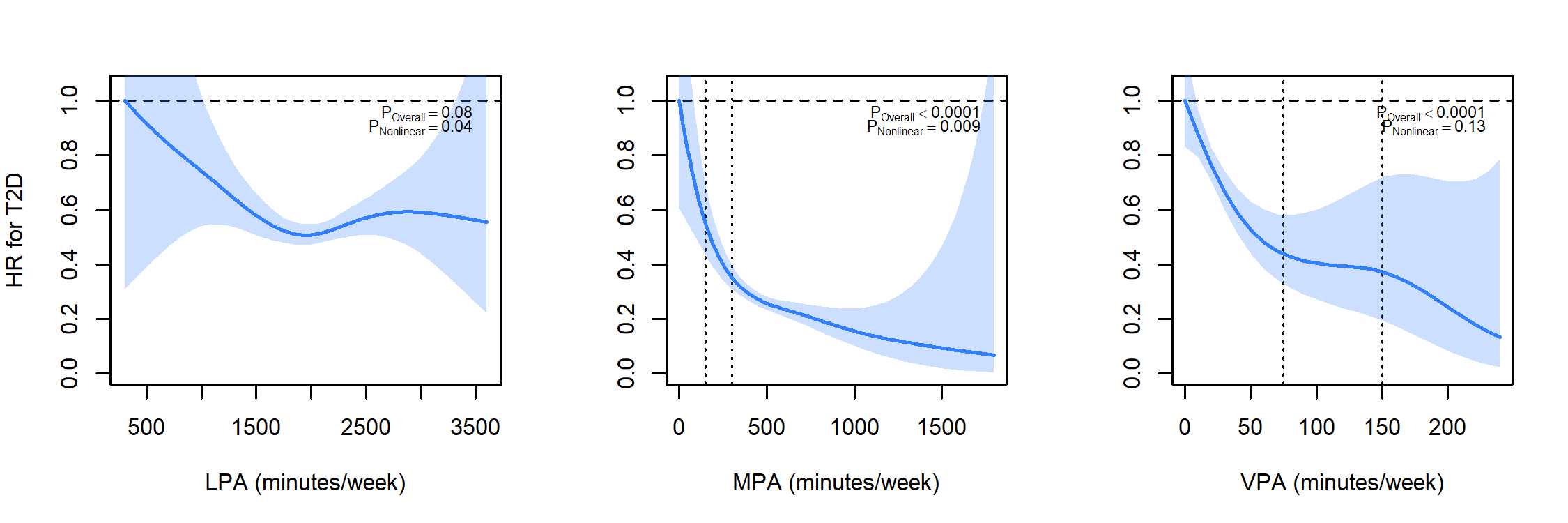

**Figure S2**. Non-linear association between physical activity domains and incident type 2 diabetes using mutually adjusted physical activity domains.

Data are presented as hazard ratios (HR) and their 95% CI. The analysis was adjusted for age, sex, deprivation, education, ethnicity, alcohol intake, and smoking status.

LPA: light physical activity; MPA: moderate physical activity; MVPA: moderate-vigorous physical activity; PA: physical activity; T2D: type 2 diabetes; VPA: vigorous physical activity


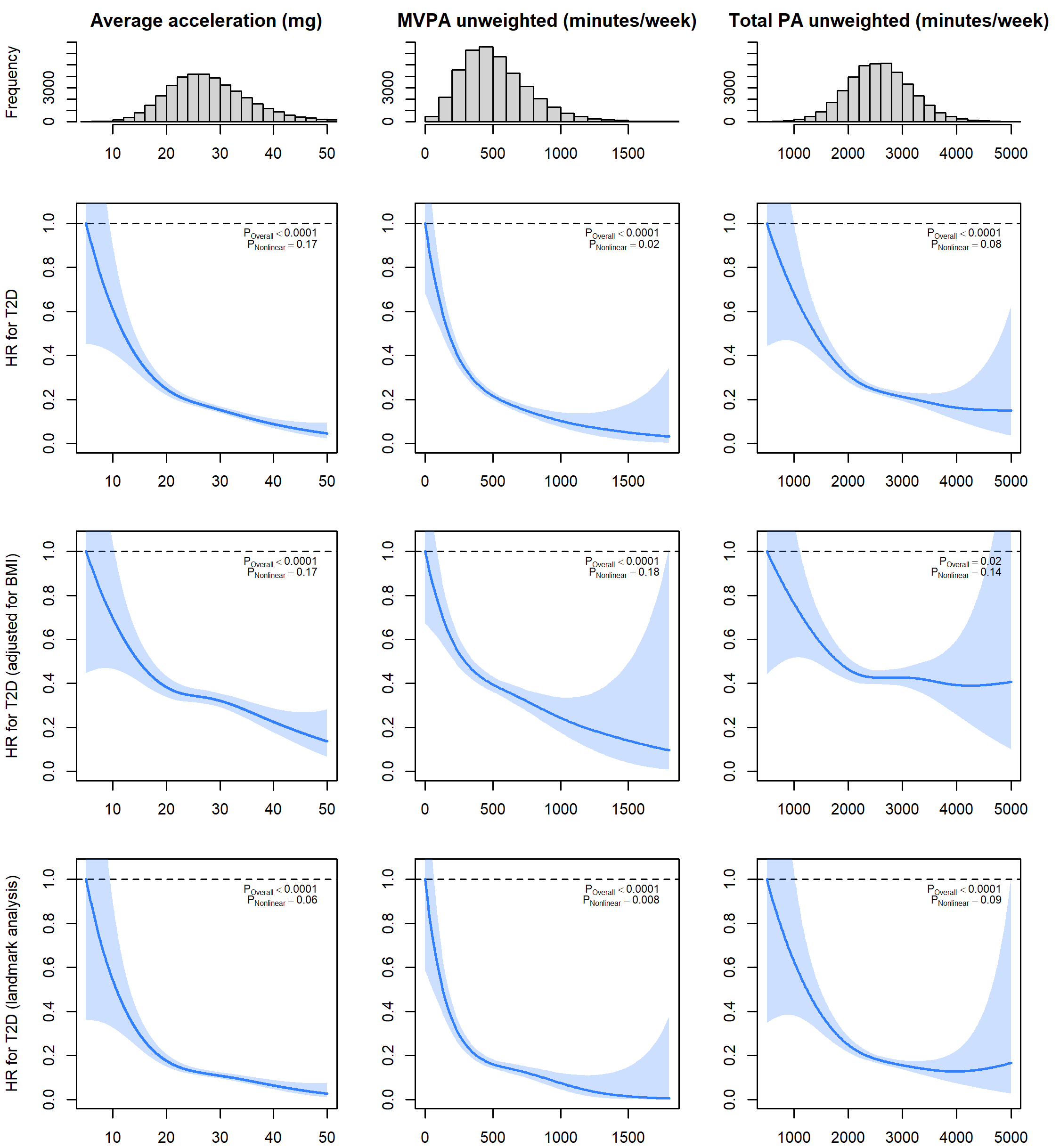


**Figure S3**. Non-linear association between unweighted physical activity domains and incident type 2 diabetes.

Data are presented as hazard ratios (HR) and their 95% CI. The analysis was adjusted for age, sex, deprivation, education, ethnicity, alcohol intake, and smoking status.

MVPA: moderate-vigorous physical activity; PA: physical activity; T2D: type 2 diabetes
